# Association of ambient temperatures with suicide attempts and violence with the future projections under climate change scenarios: a nationwide time-stratified case-crossover study in South Korea

**DOI:** 10.1186/s12889-025-21660-4

**Published:** 2025-02-05

**Authors:** Jiwoo Park, Jieun Oh, Whanhee Lee, Yeonsu Kim, Jeong Ho Park, Ho Kim, Seungsik Hwang

**Affiliations:** 1 Department of Information Convergence Engineering, Pusan National University, Yangsan, South Korea; 2 Center for Artificial Intelligence Research, Pusan National University, Yangsan, South Korea; 3 Department of Public Health Sciences, Graduate School of Public Health, Seoul National University, Seoul, South Korea; 4 School of Biomedical Convergence Engineering, Pusan National University, Yangsan, South Korea; 5 Laboratory of Emergency Medical Services, Seoul National University Hospital Biomedical Research Institute, Seoul, South Korea; 6 Department of Emergency Medicine, Seoul National University Hospital, Seoul, South Korea; 7 Institute of Health and Environment, Graduate School of Public Health, Seoul National University, Seoul, South Korea

**Keywords:** Climate change, Heat, Suicide attempts, Violence, Future projection

## Abstract

**Background:**

Both climate change and intentional injuries (suicide attempts and violence) are recognized as crucial factors that affect human health. Previous studies revealed the association between ambient temperatures and deaths due to intentional injuries but mostly about the consequences of severe events. Our study aimed to examine suicide attempts and violence incidence including mild and moderate cases with future projections, using each injury’s burden about climate change scenarios.

**Methods:**

We collected nationally representative cases of suicide attempts (8,512 cases) and violence (9,742 cases) from the Korea National Hospital Discharge In-depth Injury Survey from 2005 to 2019. We performed a two-stage analysis with a time-stratified case-crossover design to evaluate the associations of outdoor temperatures with suicide attempts and violence using historical data (2005–2019). Based on the estimated associations in the historical period, we projected the excess suicide attempts and violence attributable to ambient temperatures in the future (2020–2099) using the three shared socioeconomic pathway (SSP) scenarios.

**Results:**

We found positive associations between outdoor temperatures with suicide attempts and violence individually, and the association with violence was more linear. The excess suicide attempts attributable to temperatures in the historical period were around 11%, and it was expected to increase in all SSP scenarios (up to 14.35%). For violence, the excess risk of violence attributable to temperatures in the historical period was around 3.5%, and it was also estimated to increase in the future (up to 7.92%).

**Conclusions:**

The nationwide study about South Korea shows that there are associations between an increase in outdoor temperatures and increased risks of suicide attempts and violence, and each excess injury related to temperature is projected to grow under the SSP scenarios. Our findings might provide informative evidence for relevant action plans against climate change and intentional injuries.

**Supplementary Information:**

The online version contains supplementary material available at 10.1186/s12889-025-21660-4.

## Introduction

Mental disorders, which have gained more attention since the COVID-19 pandemic, have long been recognized as a significant social problem [1]. In particular, among mental disorders, suicide is a major and growing public health concern [2]. As of the 2019 World Health Organization (WHO) report, over 700,000 people die annually by suicide and was the fourth leading cause of death among people aged 15–29 years [2]. In addition to suicide, violence **–** which is one of the major intentional injuries **–** is another crucial public health issue. According to the WHO fact sheet regarding injuries and violence published in June 2024 [3], violence-related injuries kill 1.25 million people every year, especially in 2019, globally 6.2 deaths per 100,000 persons died due to homicide.

Moreover, suicide has been a severe social and health problem in South Korea. The Organization for Economic Cooperation and Development (OECD) reported that South Korea showed a suicide rate of 24.1 per 100,000 people in 2020. The suicide risk is the highest in the OECD countries [4]. Also, in South Korea, the homicide-related mortality rate was 0.8 per 100,000 in 2019 [5], and it was higher than in neighboring East Asian countries (0.2 and 0.3 per 100,000 in Japan and Singapore) or the OECD average [5, 6]. Further, severe violence cases have been seriously and widely addressed in mass and social media in South Korea because it is recognized as a critical factor that destroys human well-being, social justice, and quality of life [7]. Therefore, suicide and violence should be studied in-depth and timely, concurrently.

Numerous studies have examined the risk factors for suicide and violence including socioeconomic factors, individual factors, and even genetics [8]. In addition, rich environmental studies also have consistently reported that ambient temperature is one of the important risk factors for intentional injuries covering suicide [9] and violence [10]. Historically, the hypothesis that exposure to high temperatures can increase aggression through increases in moods associated with discomfort, impulsivity, and frustration has been suggested [11], and multiple epidemiological studies have identified this hypothesis. A multi-country study (12 countries) in 2019 reported that higher ambient temperature was related to increased suicide risk (a linear relationship) in most countries, except for some northeast Asian countries (including South Korea) showing an attenuation in suicide risk in extremely high temperatures [12]. A recent study in South Korea also reported a nonlinear but increasing association between summer temperatures and violent crime incidence [13]. As with many other countries, South Korea is experiencing the impacts of climate change, with average temperatures over the past 30 years having risen by 1.4 °C compared to the early twentieth century [14] and projections indicating a further increase of up to 7.0 °C by the late twentieth century [15]. This underscores the importance of examining temperature-related burdens in the context of climate change.

Nonetheless, previous studies on the temperature and suicide/violence relationships have several limitations. First, most of the existing studies investigating this topic used suicide and homicide data [12, 16, 17], thus there could be knowledge gaps regarding the impacts of temperatures on mild or moderate suicide attempts and violence that were not linked to deaths. Second, due to the limited monitoring and mortality data, many studies have included selected areas with temperature monitoring stations with a sufficient sample size for statistical analyses (suicide and homicide counts) [12, 18], mostly metropolitan or urban areas satisfied these conditions. Therefore, selection biases can exist regarding the limited areas, especially if the nationwide association should be assessed for the national public health policy. Lastly, to our knowledge, even though climate change is one of the most important global challenges of the twenty-first century affecting human health and well-being, only a few studies are estimating the future impacts of temperature on intentional injuries under climate change [19].

Therefore, to address these gaps in knowledge, this study aims to assess the nationwide risks of temperatures on attempts of the two intentional injuries (suicide attempts and violence), which allows us to provide wider information on suicide and violence than that from the mortality data covering only severe cases. This study used the national population-representative survey data covering all districts in South Korea, provided by the Korea Disease Control and Prevention Agency (2005–2022). Further, using the risk estimates from the historical data, we performed a projection study to estimate the future excess risks of suicide attempts and violence attributable to temperatures using the recent climate change scenarios. Subgroup analyses were also conducted to identify the high- and low-risk populations.

## Methods

### Data on suicide and violent attempts

We collected and used national data on discharged injuries from 2005 to 2019 in South Korea from the Korea National Hospital Discharge In-depth Injury Survey [20], which is officially operated and provided by the Korea Disease Control and Prevention Agency annually to investigate the national status regarding all types of injuries (both intentional and unintentional) with relevant statistics. This survey annually sampled 150,000 to 300,000 people (the number has increased over the years) discharged from the general hospital, and the target population is all people discharged from the general hospital residing in South Korea from 2005. Also, to get population representativeness, the survey adopted the two-stage stratified-cluster systematic sampling method, based on regions, age-sex structures, and the number of hospital beds in the selected hospitals. Theoretically, the entire general hospital should be a target for the survey; however, due to practical reasons, the survey targeted general hospitals with 100 beds or more. Among the general hospitals with 100 beds or more, the survey sampled 250 hospitals using the Neyman allocation method [21] based on the number of hospital beds of each hospital. Among survey items, the diagnostic and damaged extraneous code was based on the Korean Standard Classification of Diseases version 8 (KCD-8th) and International Classification of Diseases, Ninth Revision, Clinical Modification (ICD-9-CM) Vol.III. The injury was defined as the S00-T98 code (certain other consequences by injury, addiction, and externalities) by KCD-8th using the main diagnosis or sub-diagnosis. Each recorded case includes information about the patient’s sex, age, residential district, date of hospitalization, and injury mechanisms (transport accident, drowning, poisoning, etc.).

In this study, from the survey data, we obtained and constructed two different datasets for each case of hospital visits: 1) the dataset for suicide attempts, and 2) the dataset for violence. As the survey includes medical records-based information on the intentionality and types of intentional injuries (suicide, violence, poisoning, etc.), we used relevant information to define cases of suicide attempts and violence, individually. Suicide attempts are defined as injuries coded as X60-X84, using KCD-8th, including failure of suicide attempts, retrying suicide, cutting oneself with a knife with the intention of suicide, jumping into a running car, or from a high place, addiction with a clear intention of suicide, etc. Violence is defined as interpersonal violence and is coded as X85-Y09 from KCD-8th. It includes being punched by a person or beaten with a blunt instrument, or having been raped, etc., except for violence under legal mediation or caused by war and civil conflict.

### Historical temperature and precipitation data

We collected district-level daily mean temperature and sum precipitation data from 2005 to 2019 across 250 districts (si-gun-gu, with a median area size [397 km^2^] of approximately 1.7 times that of a ZIP code [233 km^2^] in the United States) in South Korea from Google Earth Engine ERA5-Land. In this procedure, we used the daily mean weather variables for each district by averaging the ERA5-Land values of the grid cells with each grid’s centroid points inside the boundary of that district [22]. After that, the district-specific daily weather data were linked to each discharge injury case based on their residential address and hospital visit dates.

### Projected temperature data under climate change scenarios

We collected the Coupled Model Intercomparison Project phase 6 (CMIP6) general circulation models (GCMs) and calculated the future district-level daily mean temperature data from 2020 through 2099 [23]. The CMIP6 employed the shared socioeconomic pathways (SSPs) [24], which is a new scenario framework based on socioeconomic trajectories. The SSP scenarios describe plausible future societal developments, including changes in population, economy, and energy use. These are used in conjunction with Representative Concentration Pathways (RCPs), which describe different radiative forcing trajectories.

Specifically, we obtained the daily projected temperature series for the historical (2005–2019) and projected (2020–2099) periods under three SSP scenarios (SSP2-4.5, SSP3-7.0 and SSP5-8.5) in six GCMs (ACCESS-CM2, CanESM5, CESM2, CNRM-CM6-1, CNRM-ESM2-1, KACE-1–0-G). SSP2-4.5 means that the degree of climate change mitigation and socioeconomic development is assumed to be in the middle stage. SSP3-7.0 assumes a social structure that is passive in climate change mitigation policies and is vulnerable to climate change due to slow technological development. The most serious scenario among these is SSP5-8.5 which assumes that the use of fossil fuels is high and indiscriminate development centered on urban areas will be expanded by focusing on the rapid development of industrial technology. All the daily mean temperatures were extracted from grid cells corresponding to the coordinates of each district and recalibrated using the observed series, following the methodology suggested by Hempel et al. [25]. These approaches are the standardized process used widely in previous studies on temperature-related health risk projections [26, 27].

### Statistical analysis

#### Estimation of the observed exposure–response relationships

First, this study adopted the time-stratified case-crossover design, a study method that considers oneself as a control, only having the date about the same year, same month, and the same day. It can control time-invariant and time-varying confounders like long-term trends, seasonality, and the day-of-the-week effect, showing the effect only in the short term. As a result, 1:3 or 1:4 matching was made in every case. We made a separate time-stratified case-crossover dataset for each outcome (suicidal attempt and violence).

Then, for each case-crossover dataset, two-stage analyses were conducted [12, 28]. In the first stage, we performed a conditional logistic regression to estimate the temperature-related injury risk for each of the 16 provinces. At this step, due to the insufficient sample size from the district level, we had to consider the province level, which is a higher regional scale. To apply the regional difference in temperature distributions and exposure–response curves, we aggregated our 250 districts into 16 provinces. The distributed lag nonlinear model was used for the conditional logistic model to consider temperature’s nonlinear and delayed roles [26]. The exposure–response function was modeled using a natural cubic spline function with two internal knots at the 33.3th, and 66.7th percentiles of the province-specific temperature distributions. Considering that our intentional injuries usually occur as acute events, we adopted a lag period of 0–2 days, which previous studies have widely validated [9, 12]. The lag-response function was modeled with a natural cubic spline with an intercept and one internal knot. Considering environmental time-varying confounders, we adjusted the current-day precipitation measured from the ERA5-Land.

In the second stage, we pooled the first-stage estimates to provide nationwide and province-specific estimates using a multivariate meta-regression approach. For more information about the two-stage analysis, refer to Supplementary Materials (Supplementary Methods—Observed exposure–response relationships).

Also, to investigate the sub-populations and potential effect modifications, we repeated the main analysis by sex, and age groups (aged less than 65 years / aged 65 or older).

#### Projection of excess discharge injury in the future

We respectively estimated the excess suicide attempts and violence attributable to ambient mean temperature in the future, under different SSP scenarios, using the daily series of modeled historical mean temperatures and the observed injury. There are not enough studies on the relationship between the future population and the number of intentional injuries, also to simplify our result interpretations, we followed previous studies' assumptions and methodological approaches [26, 27], assuming there are no changes in baseline population and adaptation to extreme temperature. Thus, we averaged the observed counts of each suicide attempt and violence for every day of the year from 2005 to 2019 and applied it from 2005 to 2099.

Then, we used the province-specific lag-cumulative temperature-suicide attempts and temperature-violence associations to calculate odds ratios (ORs) corresponding to each day’s temperature, setting the reference point as the 25th percentile of temperature distribution. And then, we calculated the daily attributable number and fraction of suicide attempts and violence, using the daily attributable fraction formula: 1-(1/OR_t_). The daily attributable number was calculated as the multiplication of the daily attributable fraction and the daily number of each outcome. The sum of daily attributable numbers from all days in the series represents total excess injuries attributable to temperatures lower or higher than the reference. In Supplementary Materials (Table S1), we display the reference temperature for each province.

Also, the attributable fractions (%) due to temperatures were computed by dividing the sum of the projected attributable numbers by the total counts of suicide attempts and violence, respectively [26, 27]. For the future projected period (2020–2099), we aggregated the excess number of suicide attempts and violence by decades for each SSP scenario (SSP2-4.5, SSP3-7.0, and SSP5-8.5) covering all of South Korea.

Finally, to address uncertainty in both the estimation of the exposure-lag-response associations and six GCMs, the attributable numbers by temperature on each suicide attempt and violence were estimated using the Monte Carlo simulation [26, 27]. For each GCM model, we generated 1,000 samples of Best Linear Unbiased Prediction (BLUPs) assuming the multivariate normal distribution. Then, as GCM-ensemble estimation, point estimates (averages) and 95% empirical confidence intervals (eCIs) for the attributable number and fraction of outcomes were calculated across the six GCM models and used as the main results in this study. Additional information about the projection procedure is represented in the Supplementary Materials (Supplementary Methods – Projection of excess severe injury in the future).

#### Sensitivity analyses

We did several sensitivity analyses about modeling specifications by altering the knots in the exposure–response function (the 25th, 50th, and 75th percentiles and the 10th, 75th, and 90th percentiles of temperature distributions) and extending the lag period to three and seven days.

## Results

Table 1 shows the descriptive statistics on outcomes during the study period related to the Korea National Hospital Discharge In-depth Injury Survey (2005–2019), individually. Total counts of suicide attempts and violence were 8,512 and 9,742, each. For suicide attempts, 44.3% were males, and 79.2% were individuals aged less than 65 years. In violence cases, 69.8% were males and 94.6% occurred in people aged less than 65 years.

**Table 1:** Summary statistics on data on suicide attempts and violence (2005–2019)

**Outcome**		**Populations**	**N**	**(%)**
**Suicide Attempts**		Total	8512	100
	**Sex**	Males	3767	44.3
		Female	4745	55.7
	**Age**	0–19 years	555	6.5
		20–39 years	2935	34.5
		40–64 years	3250	38.2
		65–79 years	1255	14.7
		80 years +	517	6.1
**Violence**	**Total**	Total	9742	100
	**Sex**	Males	6803	69.8
		Female	2939	30.2
	**Age**	0–19 years	1612	16.5
		20–39 years	3763	38.6
		40–64 years	3840	39.4
		65–79 years	456	4.7
		80 years +	71	0.7

**Fig. 1:**
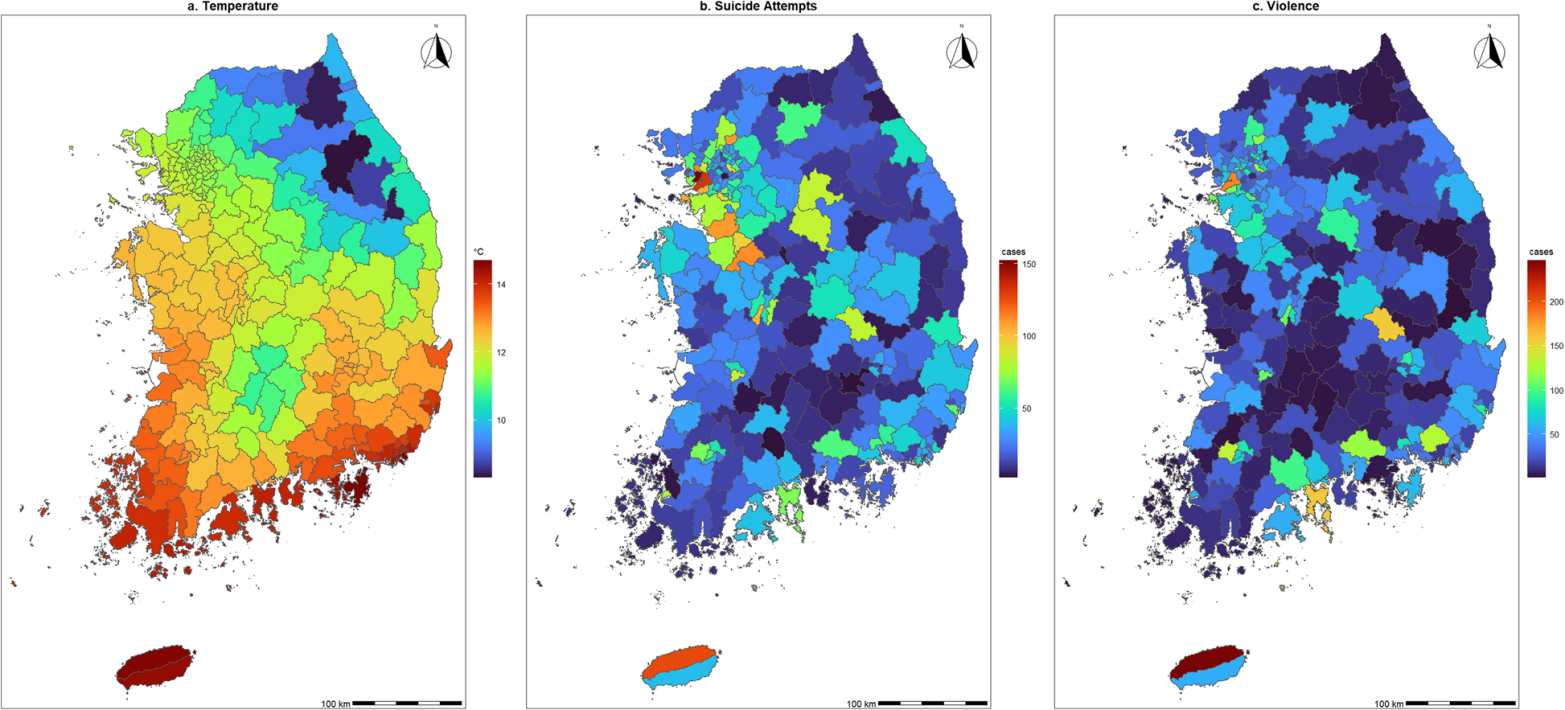
Geographical distributions of average ambient temperature by district, counts of suicide attempts, and counts of violence by the study district during the study period (2005–2019)

Figure 1 presents the geographical distributions of temperatures (**a**) and the number of cases of suicide attempts (**b**) and violence (**c**) in South Korea during the historical period (2005–2019). The boundary of Fig. 1 shows the study district, which consists of 250 districts.

Table 2 shows the temporal trends of the average temperatures in historical (2005–2019) and future projected periods (2020–2099) under three different SSP scenarios. The average temperature difference between the historical periods and the 2090s is 2.66 °C, 4.43 °C, and 5.83 °C for SSP2-4.5, SSP3-7.0, and SSP5-8.5, respectively.

**Table 2:** Average temperatures by 10 years from the historical period (2005–2019) to the 2090s by SSP scenarios. SSP: Shared Socioeconomic Pathways

Period	SSP2-4.5	SSP3-7.0	SSP5-8.5
Historical period (2005–2019)	12.79 °C	12.83 °C	12.73 °C
2020–29	13.51 °C	13.62 °C	13.50 °C
2030–39	13.82 °C	13.91 °C	13.98 °C
2040–49	14.29 °C	14.45 °C	14.78 °C
2050–59	14.69 °C	14.80 °C	15.22 °C
2060–69	14.86 °C	15.49 °C	16.05 °C
2070–79	15.27 °C	16.05 °C	16.82 °C
2080–89	15.48 °C	16.74 °C	17.71 °C
2090–99	15.45 °C	17.26 °C	18.56 °C

**Fig. 2:**
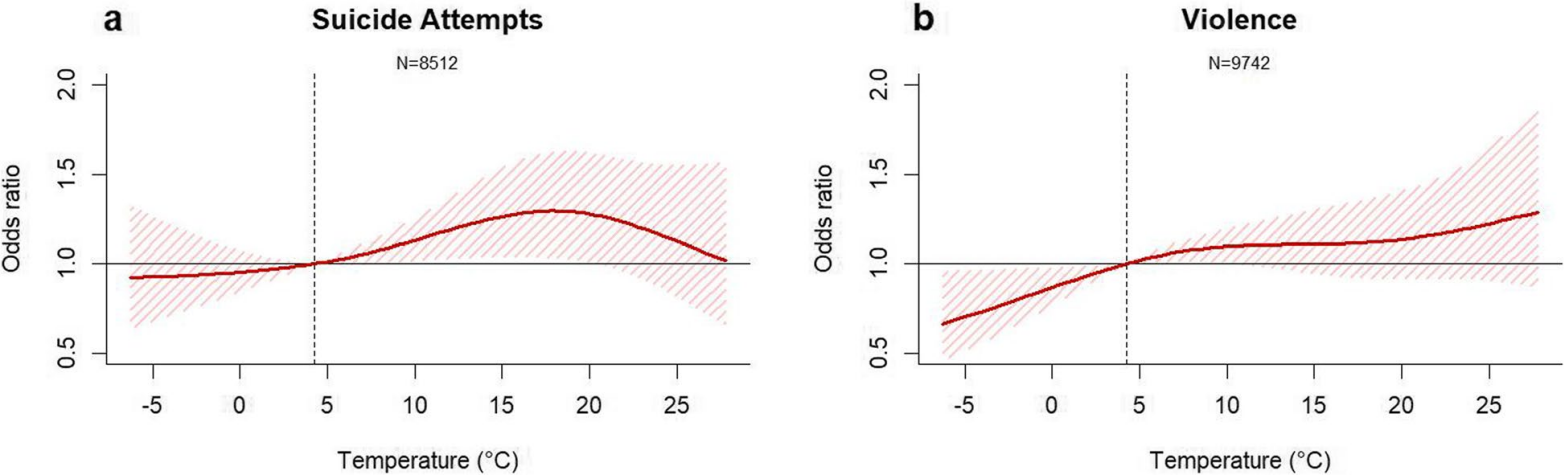
Nonlinear exposure–response curves between daily mean temperature over 0–2 lag days and outcomes: suicide attempts (**a**) and violence (**b**) cases. The shaded areas indicate 95% CIs. The dashed vertical line represents the reference, the 25.^th^ percentile of observed temperature (4.69 °C for South Korea)

Figure 2 presents the relationship between temperature and suicide attempts (a) and violence (b) in the historical period (2005–2019). For both outcomes, overall, a higher ambient temperature was associated with an increased risk of suicide attempts and violence. However, the outcomes showed a different pattern: the suicide attempt risk decreased after 18–20 °C, and the risk of violence slightly increased after 18–20 °C.

**Fig. 3:**
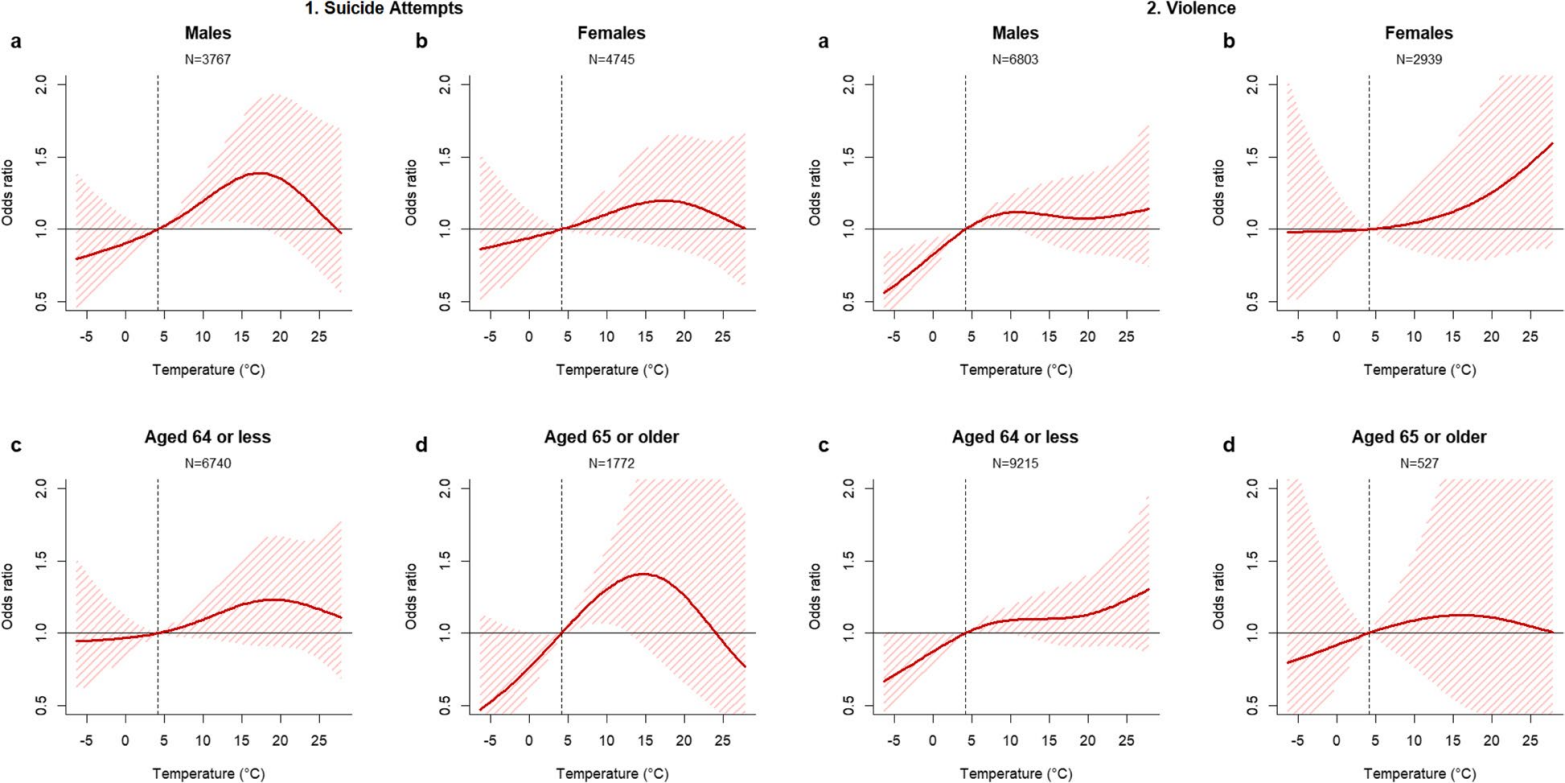
Subgroup-specific exposure–response curves between daily mean temperature over 0–2 lag days and suicide attempts. The shaded areas indicate 95% CIs. The dashed vertical line represents the reference, the 25.^th^ percentile of observed temperature (4.69 °C for South Korea)

**Fig. 4:**
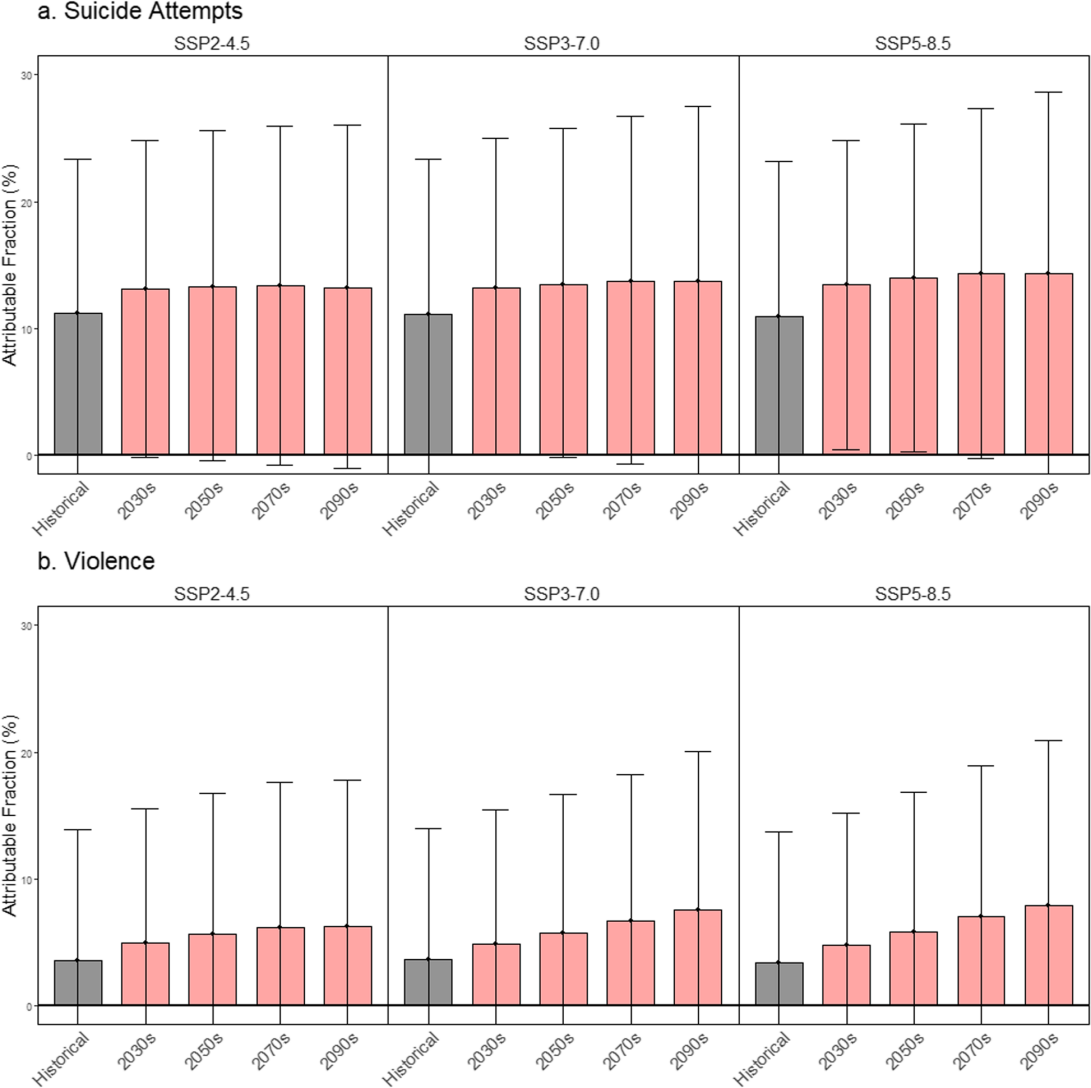
Excess risk fraction of suicide attempts (**a**) and violence (**b**) attributable to temperatures by SSP scenarios

For suicide attempts, based on the point estimates, the temperature-risk patterns are heterogeneous by subpopulations (Fig. 3–1). First, the risk slope under moderate temperatures (around 15 °C) was higher in people aged 65 or older than less than 65, while the risk at high temperatures (over 18–20 °C) was more prominent in people aged less than 65. Second, the temperature-related suicide attempt risk pattern was similar in both sexes, but it was slightly more evident in males. For violence, shown in Fig. 3–2, the risk pattern was more pronounced in people aged 64 and less and females, compared to that of people less than 65 years and males. Figure 4 shows the estimated GCM-ensemble excess risk fraction of suicide attempts (a) and violence (b) attributable to temperatures in the historical period (2005–2019) and the future period (2020–2099 by decades) by SSP scenarios. For suicide attempts, the attributable fraction due to temperature in the historical period was around 11% and was expected to increase in the future across all SSP scenarios, showing a heterogeneous amount of the increase by scenarios. First, under SSP2-4.5, the excess suicide attempts attributable to temperature will gradually increase, with the excess risk in the 2090s estimated to be 13.23% (95% eCI: -1.04, 26.06). However, in the worst mitigation scenario SSP5-8.5, the increase in the excess risk shows the fastest, and the excess risk in the 2090s is estimated at 14.35% (95% eCI: -1.76, 28.66).

For violence, the excess risk of violence attributable to temperatures in the historical period (2005–2019) was around 3.5%, which was smaller than the excess risk of suicide attempts. Also, across all SSP scenarios, the excess risks of violence attributable to temperatures are expected to increase during the twenty-first century. Under the scenario, same as suicide attempts, SSP5-8.5 shows the fastest increase in excess risk, and the excess risk estimates in the 2090s were 7.92% (95% eCI: -6.6, 20.92). The absolute number of excess suicide attempts and violence in the historical and future periods (by 10 years) is reported in Table 3. The estimated excess number of suicide attempts and violence in the 2090s is 814.49 and 514.63 cases under SSP5-85.

**Table 3:** Excess number of suicide attempts and violence attributable to temperatures over the 25th percentile of temperature distribution for each SSP scenario. SSP: Shared Socioeconomic Pathways

		**The excess number due to the temperatures**		
		**SSP2-4.5**	**SSP3-7.0**	**SSP5-8.5**
**Suicide attempts**	**Historical period (2005–2019)**	955.5(-180.5,1990.7)	945.3(-197,1989.6)	931.6(-201.4,1969.3)
	**2020–29**	733.6(-8,1394.9)	754.3(8.1,1417.2)	748.6(21.7,1387.6)
	**2030–39**	742.4(-11,1410.6)	750.7(-1.8,1419.4)	761.6(21.7,1411.2)
	**2040–49**	747.2(-20.5,1438.8)	760.5(-1.7,1447.3)	788.8(20.7,1466.9)
	**2050–59**	752.3(-26.6,1453)	762.7(-12,1461.5)	791.8(11.8,1481.6)
	**2060–69**	750.7(-33.6,1460.7)	780.5(-18.5,1498.3)	805.3(-0.5,1521.9)
	**2070–79**	756.2(-43.4,1473.9)	777.6(-40.5,1515.3)	813.9(-16.9,1554.1)
	**2080–89**	755.8(-54.6,1486.8)	781.7(-70.9,1548.5)	813.9(-54.9,1586.5)
	**2090–99**	751(-59.1,1478.6)	775.8(-109.8,1560.8)	814.5(-100,1626.4)
**Violence**	**Historical period (2005–2019)**	341.5(-788.5,1353)	350.2(-788.2,1362.4)	328.5(-804.4,1331.3)
	**2020–29**	301(-444.9,980.8)	305(-441.1,985.8)	279.2(-456,944.1)
	**2030–39**	319.6(-438.2,1010.1)	317.8(-428.8,1001.2)	308.6(-439.6,987.2)
	**2040–49**	341.7(-425.3,1047.1)	356.5(-403.5,1063.7)	354.2(-419.9,1066.8)
	**2050–59**	368.4(-416.1,1087.4)	370.4(-394,1082.7)	375.7(-402,1094.3)
	**2060–69**	376.5(-413.4,1098.5)	412.3(-383.9,1146)	418.7(-381.1,1164.4)
	**2070–79**	402(-403,1146.3)	435.3(-381.9,1183.7)	453.6(-369.2,1228.3)
	**2080–89**	411.4(-400.7,1165.7)	467.1(-395.3,1246.7)	486.2(-389.2,1294.4)
	**2090–99**	405.7(-401.6,1155.3)	489.8(-435.3,1299.8)	514.6(-428.6,1358.8)

**Fig. 5:**
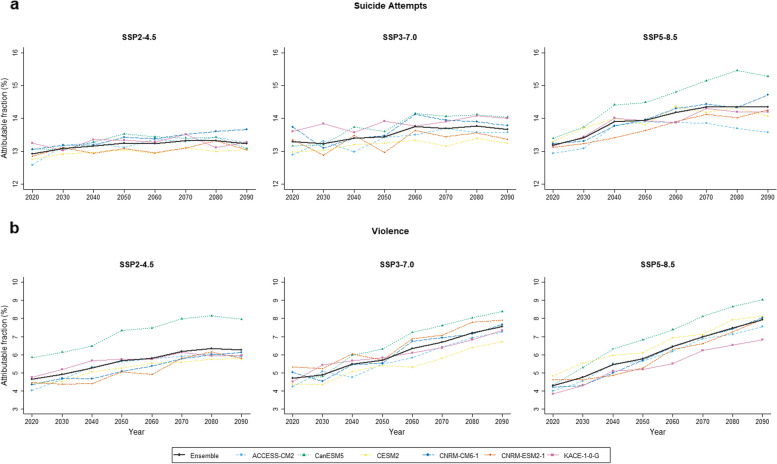
Temporal trends in excess fraction of suicide attempts (**a**) and violence (**b**) attributable to temperature under three SSP scenarios. The black point represents the mean point estimate of the percentage excess. The black vertical segments denote 95% eCIs of the percentage excess

Figure 5 represents the inter-model variabilities when six different GCM models were used to compute the excess risk of suicide attempts (a) and violence (b) attributable to temperatures. The CanESM5 model showed the highest risk estimates for both outcomes across all SSP scenarios. On the other hand, the CNRM-ESM2-1 model generally provided the lowest risk estimates across SSP scenarios; however, the model generating the lowest risk estimates was heterogeneous depending on periods and SSP scenarios. These results would highlight the necessity to consider the uncertainty among different GCM models as done in this study.

Lastly, our risk estimates in the total population remained robust in the sensitivity analyses after adjusting for different modeling specifications (Figure S1).

## Discussion

This study estimated the national excess risks of suicide attempts and violence related to ambient temperature respectively using the nationwide and population-representative survey for both outcomes. In the historical period (2005–2019), the excess risk fractions of suicide attempts and violence due to temperatures were approximately 11% and 3.5%, individually, in the total population. The excess risks of suicide attempts were generally higher in people aged 65 years or older and males, while excess risks of violence were higher for those aged less than 65 years and females. In addition, this study projected the future excess risks fractions of suicide attempts and violence due to temperatures using 6 different GCMs for three SSP scenarios. For both suicide attempts and violence, the excess risks due to temperatures were expected to increase across all GCMs and SSP scenarios, and the highest excess risks for suicide attempts (14.35%) and violence (7.92%) were observed in SSP5-8.5.

There has been a growing body of literature on the association between temperature and intentional injuries. One multi-country study investigated an inverted J-shaped, non-linear association between temperature and suicide in northeast Asian countries [12]. A study in California found that increased temperatures were associated with increased suicide and homicide mortality risk [18]. Another study in the US found a positive temperature-homicide association in some large cities [17]. However, most previous studies have targeted more severe and fatal outcomes (i.e., suicide or homicide), and the association between temperature and injuries due to suicide attempts and violence, which include mild or moderate cases, has not been sufficiently explored. Some recent studies have utilized emergency ambulance transport records and national violent crime data records [13, 29], and our findings add to this limited area of research by using survey data based on actual medical records.

There are several plausible mechanisms driving temperature-related suicide attempts and violence. Previous studies suggested that climate change, including temperature, can directly affect mental health by exposing people to trauma [30–32]. In addition, an increase in temperature can impair the function of the central nervous system through various methods such as serotonin and dopamine amount control and homeostasis, which can lead to hostility aggressive thoughts, and possibly actions [11, 31]. It could be noted that as the temperature rises, people get outside and have more social interactions, and this could increase the opportunity for interpersonal conflict [33]. However, in extreme heat, people can avoid the negative health impacts of temperatures by escaping to cooler indoor environments, such as homes or internal places [34]. Further, it has been suggested from multiple studies that extremely high temperatures can cause physical discomfort, fatigue, and dehydration, which can rather inhibit aggressive behavior [35–37]. Meanwhile, some have hypothesized that staying indoors on extremely hot days leaves people from natural surveillance of the ‘eyes on the street’, and that this lack of protection could lead to contact between motivated offenders and potential victims and increase the risk of violence [37, 38]. The mechanisms for temperature-related intentional injuries are heterogeneous and interconnected, and more research is needed to understand the specific biological and psychological pathways for the effects of temperature on suicide and assault, respectively.

As climate change progresses, it is increasingly important to understand temperature-related health outcomes and prepare for future risks. In this context of global warming, our study could have important societal implications [27]. In this study, we quantified the future burden of suicide attempts and violence due to temperatures. We found that the future burden of intentional injuries may vary under different SSP scenarios. Under the SSP2-4.5 scenario, the excess risk of both suicide attempts and violence attributable to temperatures was projected to increase and then plateau from the mid-21st century (2050–2070s). However, under the SSP3-7.0 and SSP5-8.5 scenarios, the excess risk was expected to continue increasing through the 2090s, with the largest increase under SSP5-8.5. These findings importantly suggest that the burden of intentional injuries may worsen as temperatures rise in the future and emphasize the need to take action on climate change to prevent and control intentional injuries.

This study found the variability in the temperature-related burden by type of intentional injury, with a consistently greater burden for suicide attempts compared to violence from the historical to future periods. These results are generally consistent with previous studies on the future burden of temperature-related injury deaths (i.e., suicide and homicide) [39, 40]. The exposure–response relationship curve estimated in this study might partly explain the heterogeneity by type of intentional injuries. Overall, an increase in temperatures was associated with a higher risk of suicide attempts and violence. For suicide attempts, we found an inverted J-shape association, increasing sharply at moderate temperatures and then decreasing at extremely high temperatures, as observed in previous studies [9, 12]. However, for violence, there is no consensus on the association with temperatures; some studies found an inverted U-shaped relationship [13] and others found a positive linear relationship [41, 42]. In this study, we observed that the risk of violence increased slowly at mild temperatures and then more rapidly at extremely high temperatures. Therefore, suicide attempts, which were estimated to have a higher risk in normal and moderate temperatures, were projected to have a higher temperature-related burden compared to violence. Nonetheless, this study is limited in explaining the differences in the association of temperatures with suicide attempts and violence fully; therefore, the differences should be addressed in-depth in the future, especially in the context of climate change and social or cultural heterogeneities, including cultural norms surrounding mental health, disparities in society, access to mental health services, and social support networks.

This study reveals the potential for significant increases in suicide attempts and violence rates under climate change scenarios, highlighting the severe impact of climate change on mental health. Consequently, a two-pronged approach is urgently needed: mitigation and adaptation [43]. Mitigation, primarily through greenhouse gas emission reduction, is essential to minimize the detrimental effects of climate change on mental health [9]. Adaptation requires raising awareness and recognition of these impacts among healthcare systems, policymakers, and local communities, and implementing strategies such as expanding mental health services and community-based support programs and establishing a surveillance system for continuous monitoring of temperature-related mental health outcomes. Nationally, this requires a cross-governmental, collaborative approach, including the development of early warning systems for extreme weather events, targeted support for vulnerable populations (e.g., the elderly), and the integration of mental health services into existing disaster response plans.

This study has several strengths. First, we targeted intentional injury cases, which also include nonfatal cases that have been less examined in comparison to suicide and homicide death cases. Second, to the best of our knowledge, this is the first study to estimate future burdens for suicide attempts and violence, respectively, under different climate change scenarios. Third, we applied the time-stratified case-crossover design, which allows us to control for potential time-invariant confounders. Finally, this study was based on more than 15 years of national datasets, thus the generalizability and statistical robustness could be improved than previous studies [18, 29].

Nevertheless, this study has several limitations. First, similar to previous epidemiological studies, our study used ambient temperature measured based on residential address as a proxy of individual exposure, which can lead to measurement error in exposure, although these measurement errors are likely to be random and may underestimate the risk [44]. Nevertheless, we used district-level temperatures as an exposure, because our dataset provided only district-level addresses. Therefore, exposure misclassification and measurement errors might exist in our results, and they should be interpreted carefully. Second, the projections of future burden were based on assumptions of no population change or adaptation in the exposure–response relationships, which can lead to uncertainty in estimates. Third, our data only included patients admitted to the hospital and discharged, and we could not identify patients who were not admitted or died before arriving at the hospital. Finally, there could be some time-varying confounders (i.e., health behaviors), however, these variables are unlikely to change significantly within a month and may have limited impacts on the results.

## Conclusion

This study evaluated the excess risks of suicide attempts and violence attributable to temperatures using the nationwide and population-representative survey provided by the Korean Disease Control and Prevention Agency. And estimated the future excess risks of both outcomes attributable to temperatures using different climate change scenarios. Our study results revealed that the excess risks of suicide attempts and violence attributable to temperatures were 11% and 3.5% approximately in the current period. However, even though all different GCMs and SSP scenarios are assumed, the risk estimates are expected to increase consistently. Our results could provide quantitative values to establish evidence-based action plans targeting suicide attempts and violence in the era of climate change.

## Supplementary Information

Supplementary Material 1.

## Data Availability

Discharge injury data for this study is provided by the Korea Disease Control and Prevention Agency. These data are available upon reasonable request from the In-depth Investigation of the Discharge Injury database, [https://www.kdca.go.kr/injury/biz/injury/main/mainPage.do]. The analytic R code of this study can be provided by the corresponding author upon request.

## Abbreviations

SSP: Shared Socioeconomic Pathway
ERA5-Land: ECMWF Reanalysis v5-Land
CMIP6: Coupled Model Intercomparison Project phase 6
GCM: General Circulation Model
