# Supplementary material for: Association of ambient temperatures with suicide attempts and violence with the future projections under climate change scenarios: a nationwide time-stratified case-crossover study in South Korea
Source: BMC Public Health. 2025 Feb 5;25:457. doi: 10.1186/s12889-025-21660-4 (PMC11796151; doi:10.1186/s12889-025-21660-4)
Supplement: Supplementary file 1 — Supplementary Material 1. [file 12889_2025_21660_MOESM1_ESM.docx]

**Supplementary Materials**

**Authors:** Jiwoo Park^1,2^*, Jieun Oh^3^*, Whanhee Lee^4,2^, Yeonsu Kim^3^, Jeong Ho Park^5,6^, Ho Kim^3^, and Seungsik Hwang^3,5,7^

1 Department of Information Convergence Engineering, Pusan National University, Yangsan, South Korea

2 Center for Artificial Intelligence Research, Pusan National University, Yangsan, South Korea

3 Department of Public Health Sciences, Graduate School of Public Health, Seoul National University, Seoul, South Korea

4 School of Biomedical Convergence Engineering, Pusan National University, Yangsan, South Korea

5 Laboratory of Emergency Medical Services, Seoul National University Hospital Biomedical Research Institute, Seoul, South Korea

6 Department of Emergency Medicine, Seoul National University Hospital, Seoul, South Korea.

7 Institute of Health and Environment, Graduate School of Public Health, Seoul National University, Seoul, South Korea

*Contributed equally as the co-first authors

**Corresponding author**

Seungsik Hwang, Department of Public Health Sciences, Graduate School of Public Health, Seoul National University, 1 Gwanak-ro, Gwanak-gu, Seoul, 08826, Republic of Korea, E-mail: cyberdoc@snu.ac.kr

**Supplementary Methods**

***Observed exposure-response relationships***

The analyses follow a two-stage approach to derive the province-specific injury risk related to temperature. In the first stage, we used a time-stratified case-crossover design with conditional logistic regressions to estimate the temperature-related injury risk for each of the 16 provinces. The time-stratified case-crossover design compares the exposure to the daily mean temperature on the day of injury occurred with the exposure during control periods on an individual level. In this study design, the same days of the week in the same calendar month were selected as control periods, and therefore, each case had three to four controls. This approach is effective in controlling for time-varying confounders, including long-term trends, seasonality, and the day-of-the-week effect, and time-invariant confounders, including sex, age, economic conditions, and lifestyle (Barnett and Dobson, 2010; Tobias et al., 2024).

Specifically, we applied conditional logistic regression to fit the association between temperature and two injuries: suicide attempt and violence. The formula is as follow:

$$\mathrm{Logit}\left( P\left( Case=1 in stratum i | T_{obs} \right) \right)=\alpha_{\mathrm{stratum} i}+cb\left( T_{obs} \right)+ns\left( \mathrm{rh} \right)+ns(\mathrm{PM}_{2.5})$$

where $P\left( Case=1 in stratum i | T_{obs} \right)$ is the conditional probability of being a case of injury in the *i*th stratum given the observed temperature; $\alpha_{\mathrm{stratum} i}$ is an indicator variable of stratum *i*; $cb\left( T_{obs} \right)$ represents a matrix produced by cross-basis function for daily observed mean temperature to model a distributed lag nonlinear model (DLNM) (Gasparrini et al., 2010). In this study, the exposure-response function was modeled using a natural cubic spline function with two internal knots at the 33.3^th^, and 66.7^th^ percentiles of the province-specific temperature distributions. Considering that injuries usually occur as acute events, we adopted a lag period of 0–2 days based on a previous study (Kim et al., 2019). The lag-response function was modeled with a natural cubic spline with an intercept and one internal knot; $ns$ is a natural cubic spline function, and we included total precipitation as linear to account for environmental time-varying confounders. Then, we reduced the high dimensional estimates to obtain the temperature-injury association cumulated over the lag period for each province.

In the second stage, we pooled the province-specific reduced estimates to the nationwide level ($\eta^{*}$), using a multivariate meta-regression approach. We then derived the best linear unbiased prediction (BLUP; $\eta_{b}^{*}$) for each of the 16 provinces to stabilize the estimates by allowing areas with a small number of cases to use information from larger populations (Gasparrini et al., 2012). We repeated the main analysis described earlier for stratified analysis by sex and age, using 65 years old.

***Projection of excess injury in the future***

We assessed the excess injury number ($D_{attr}$) attributable to temperature in Korea in the future under different SSP scenarios, using the daily series of modeled temperatures and the observed injury. For each province in Korea, we used the lag cumulative odds ratio corresponding to each day’s temperature, to calculate the daily attributable number of injuries in the next two days, using the 25^th^ percentile of temperature distribution as the reference, as follows:

$$D_{attr}=(1-exp(-\left( \eta_{b}^{*}\times T_{mod} \right))\times D_{obs}$$

where $T_{mod}$ is a modeled and recalibrated temperature during the historical (2005–2019) and projected (2020–2099) periods; $\eta_{b}^{*}$ is the province-specific BLUPs with the reference of the 25^th^ percentile of temperature distribution; $D_{obs}$ refers to the average observed counts for each day of the year, and therefore, the projected temperature-related impact must be interpreted under a scenario with no change in baseline population and adaptation to extreme temperature. The sum of attributable numbers ($D_{attr})$ from all days in the series represents total excess injuries attributable to temperatures lower or higher than the reference for each injury case: suicide attempt, and violence.

Next, we used the combination of GCMs and SSPs and aggregated the excess number by SSP scenarios for each of the 16 provinces and the entirety of Korea. We also aggregated the excess number by decade, covering the historical period (2005–2019) and projected period (2020–2099). The attributable fraction of each injury was calculated by decade by dividing the projected number by the total number.

Finally, we conducted the Monte Carlo simulations to address uncertainty in both the estimation of the exposure-lag-response associations and projections across six GCMs. Specifically, we generated 1,000 samples for each GCM model and obtained the point estimates (i.e., mean and median) with 95% empirical confidence interval (eCI) for temperature-related risks from the results across all six GCM models. All these procedures were based on the methodological approach in previous studies (Lee et al., 2020; Vicedo-Cabrera et al., 2019).

**Table S1. The reference for the exposure-response relationships was identified at the 25^th^ percentile of temperature distribution, for each of the 16 provinces.**

| **Provinces** | **The 25^th^ percentile of temperature (°C)** |
| --- | --- |
| South Korea | 4.69 |
| Seoul | 4.35 |
| Busan | 7.91 |
| Daegu | 5.91 |
| Incheon | 4.01 |
| Gwangju | 5.76 |
| Daejeon | 4.53 |
| Ulsan | 7.41 |
| Gyeonggi | 3.49 |
| Gangwon | 2.71 |
| Chungcheongnam-do | 2.92 |
| Chungcheongbuk-do | 3.81 |
| Jeollanam-do | 4.12 |
| Jeollabuk-do | 5.85 |
| Gyeongsangnam-do | 4.34 |
| Gyeongsangbuk-do | 6.08 |
| Jeju | 10.14 |

**Figure. S1. Sensitivity analyses**

**
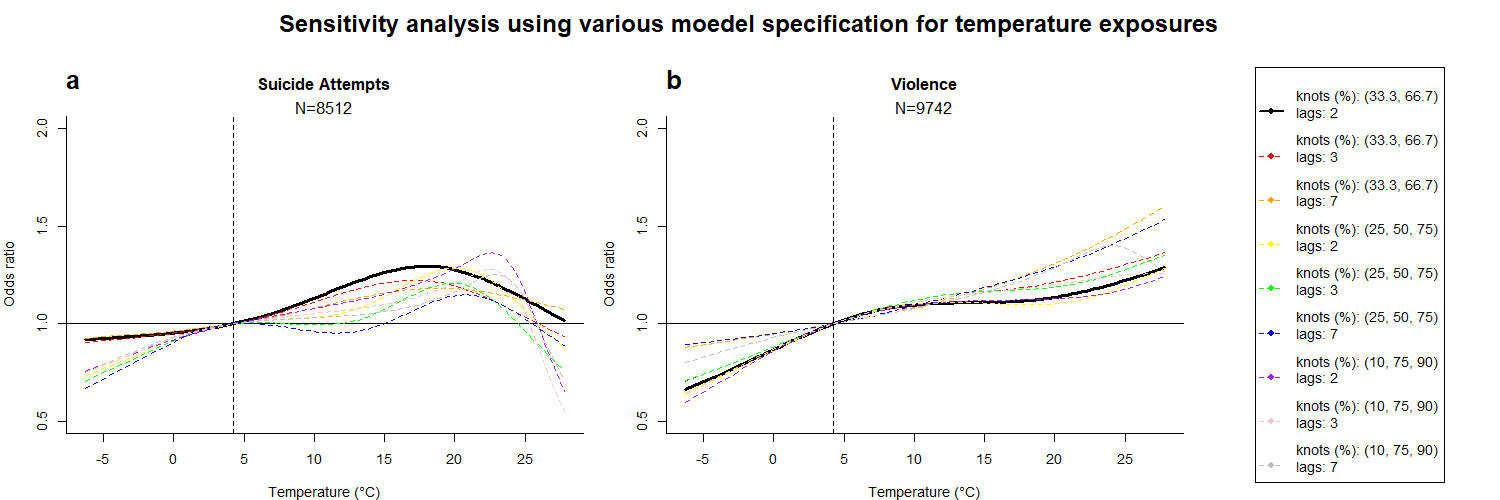
**

**References**

Barnett, A. G., Dobson, A. J., 2010. Analysing seasonal health data. Springer.

Gasparrini, A., et al., 2010. Distributed lag non‐linear models. Statistics in medicine. 29**,** 2224-2234.

Gasparrini, A., et al., 2012. Multivariate meta‐analysis for non‐linear and other multi‐parameter associations. Statistics in medicine. 31**,** 3821-3839.

Kim, Y., et al., 2019. Suicide and ambient temperature: a multi-country multi-city study. Environmental health perspectives. 127**,** 117007.

Lee, W., et al., 2020. Projections of excess mortality related to diurnal temperature range under climate change scenarios: a multi-country modelling study. The Lancet Planetary Health. 4**,** e512-e521.

Tobias, A., et al., 2024. Time-stratified case-crossover studies for aggregated data in environmental epidemiology: a tutorial. International Journal of Epidemiology. 53**,** dyae020.

Vicedo-Cabrera, A. M., et al., 2019. Hands-on tutorial on a modeling framework for projections of climate change impacts on health. Epidemiology. 30**,** 321-329.
